# *Schistosoma mansoni* and other helminthes infections at Haike primary school children, North-East, Ethiopia: a cross-sectional study

**DOI:** 10.1186/s13104-017-2942-9

**Published:** 2017-11-21

**Authors:** Daniel Getacher Feleke, Solomon Arega, Mulien Tekleweini, Kegnitu Kindie, Alemu Gedefie

**Affiliations:** Department of Medical Laboratory Science, College of Medicine and Health Sciences, Wollo University, Dessie, Ethiopia

**Keywords:** *Schistosoma mansoni*, Helminthes infection, Prevalence, Ethiopia

## Abstract

**Objectives:**

Schistosomiasis and soil-transmitted helminthes infections are among the widely distributed infections worldwide. In Ethiopia, parasitic helminthic infections and schistosomiasis are among the most predominant causes of outpatient morbidity. Hence there is still lack of epidemiological information in North-Eastern Ethiopia, this study aimed to determine the prevalence and associated factors of *Schistosoma mansoni* and other helminthes infections at Haike primary school children, Haike, North-East Ethiopia.

**Results:**

The overall prevalence of *S. mansoni* and other helminthes infections using formol-ether concentration technique was 85/279 (30.5%). *Schistosoma mansoni* was the dominant parasites as detected by both direct wet mount and formol-ether concentration technique with 44/52 (84.6%) and 65/85 (76.5%) respectively. Chi square test showed significant association between parasitic infections and age of the school children (p = 0.003). The binary logistic regression analysis was showed strong statistical association (p = 0.00) between swimming habit and parasitic infections (AOR = 6.61, 95% CI 3.31–13.12). Family used lake as source of water showed statistically significant association (AOR = 5.35, 95% CI 2.97–12.32). Furthermore, those who get water from river (AOR = 1.24, 95% CI 0.33–4.66) were more likely to be infected with *S. mansoni* and geo-helminthes than those who used tap water.

**Electronic supplementary material:**

The online version of this article (10.1186/s13104-017-2942-9) contains supplementary material, which is available to authorized users.

## Introduction

Schistosomiasis and soil-transmitted helminthes infections are among the widely distributed infections worldwide [1]. According to World Health Organization (WHO) estimate 1.45 billion people are infected with *A. lumbricoides*, 1.3 billion with hookworms and 1.05 with *T. trichiura* [2]. In sub-Saharan African countries, up to 250 million people are estimated to be infected with at least one or more intestinal nematodes [3]. School children are at high risk of intestinal parasitic infections due to poor hygiene, low immune status, overcrowding, close contact with soil and to each other, lack of latrine, and low provision of schools [1, 2].

Schistosoma infected an estimated 207 million people and more than 700 million people are at risk worldwide [4]. Schistosomiasis is one of the most prevalent and important parasitic diseases in developing countries [5]. An estimated 660 million people were at risk of schistosomiasis in Africa, it accounts for 85% of the global at-risk estimate [2]. School children who are under 15 years of age and living in endemic areas constitute a high risk group and are the worst affected by schistosomiasis [6].

People can be infected with schistosoma when they are in contact with infested fresh water during their normal daily activities of fishing, farming, swimming, washing, bathing, recreation, and irrigation [7].

In Ethiopia, parasitic helminthes infections are the second most predominant causes of outpatient morbidity in the country [1]. Schistosomiasis is also common in different geographical area and, it is a common disease in the northern region as compared to south and south west regions of Ethiopia [2, 8]. In the last decade, an estimated number of 29.89 million people were at risk, of which 4 million were infected by intestinal schistosomiasis in the Ethiopia [9]. Schistosomiasis affected more males than females [10]. Although different studies conducted, there is still lack of epidemiological information in North-Eastern Ethiopia. Therefore, this study aimed to determine the prevalence and pre-disposing factors of *Schistosoma mansoni* and other helminthes infections among Haike primary school children Haike, North-East Ethiopia.

## Main text

### Methods

#### Study area and participants

This cross-sectional study was conducted at Haike primary school from April to June 2017. Haike town is located about 430 km from Addis Ababa, North-East of Ethiopia (Additional file 1). It has an altitudinal range of 1480–1900 m above sea level. The climate is moderately hot with an annual average temperature ranging from 15 to 21 °C and annual rainfall of approximately 1030 mm. According to 2007 national census conducted by central statistical agency (CSA) of Ethiopia; the population of Haike town was estimated about 25,000. The town has two main fresh water bodies; these are Lake Haike covering an area of 23 km^2^ and Ketie permanent stream. Haike primary school is very close to Ketie stream; it has 500 m distance.

#### Sample size determination

The sample size was determined using single population proportion formula by taking 23.9% prevalence of *S*. *mansoni* infection [11]. The sample size was calculated as follows:

$${\text{N}}\,{ = }\,\frac{{{\text{Z}}^{2} {\text{P}}\left( {1 - {\text{P}}} \right)}}{{{\text{d}}^{2} }}$$

where N = sample size, P = prevalence estimate 23.9% (0.239).

$${\text{N}}\,{ = }\,\frac{{\left( {1.96} \right)^{2} \times 0.239\,\left( {1 - 0.239} \right)}}{0.05}$$

d = marginal error 5% = 0.05; Z = significant value 95% = 1.96; N = 279.

#### Stool specimen collection and processing

Two-hundred and seventy nine students were selected from the students class rosters using simple random sampling method after a quota was allocated for each grade and each class room. School children who are willing and didn’t take any anti-helminthic drugs within 4 weeks from the time of sample collection were included in this study.

A pre-tested questionnaire based on known risk factors was developed and modified. Data were collected by medical laboratory technologists and to ensure reliable information, the children were interviewed in Amharic which is their mother tongues.

The interview included information such as age, family size, source and storage conditions of drinking water, and existence of latrines in their homes. At the time of conversation, interviewers also inspected whether the fingernails of the students were trimmed and their foot wear. Study participants were given a dry, clean, leak-proof container with applicator stick and were told to directly pass the stool to the container. The stool cup was labeled with student’s identification card number, date and grade level of the students. Approximately 5 g stool specimen was collected from each student by medical laboratory technologists who were selected and trained for the purpose. Too small or mixed specimen with soil was not accepted. In this case the study participants were requested to bring the stool sample again. The stool sample was examined microscopically using direct wet mount in the study site by two medical laboratory technologists. The rest stool samples were preserved using 10% formalin and transported to Wollo University medical parasitology laboratory. Formal-ether concentration technique was performed by another two laboratory technologists who are unaware of the direct wet mount result. All positive samples were confirmed by a senior laboratory technologist who had training and is certified. He had also participated in national intestinal schistosoma and intestinal parasites surveys.

#### Direct smear examination for stool samples

About 1–2 mg of stool sample was emulsified in a drop of normal saline (0.85% NaCl) on the center of the slide. A cover-slip was placed on the sample and was examined systematically the entire saline preparation with 10× and 40× objective lenses.

#### Formol ether concentration technique

Most types of worm eggs including schistosomes eggs can be recovered by formol ether concentration technique. Using a stick, an estimated 1 g (pea-size) of representative faeces was emulsified in about 4 ml of 10% formol water contained in a screw-cap tube. Then further 3–4 ml of 10% v/v formol water was added and mixed well by shaking. The emulsified faeces were sieved using gauze and the suspension was transferred to a centrifuge tube. Then 3–4 ml of diethyl ether was added. The tube was caped and mixed for 1 min. It was immediately centrifuged at 3000 revolution per minute (rpm) for 1 min. After centrifugation, the fecal debris was separated in a layer between the diethyl ether and the 10% formal-saline layers. A fecal debris layer was loosened by wooden stick and the tube was rapidly inverted to discard the ether, faecal debris, and formol water. The bottom of the tube was tapped to re-suspend and mix with the sediment. Finally, the sediment was transferred to a slide and covered with a cover glass. Then the preparation was examined microscopically using the 10× and 40× objective lenses.

#### Data analysis

The data were entered in Microsoft Excel spreadsheet, exported and analyzed using SPSS version 20. Chi square test for the association between dependent and independent variables was used and the results were summarized in tables and figures. Binary logistic regression was done to investigate the relationship between the dependent and explanatory variables. p < 0.05 were considered statistically significant in all comparisons.

#### Ethical consideration

Ethical clearance was obtained from the institutional review board of Wollo University, College of Medicine and Health Sciences. Consent form was used to ask students’ or guardians, willingness. School children who were infected with *S. mansoni* and other helminthes infection helminthes were treated with the respective drugs at the nearby health center by communicating with the school teachers and the students’ families.

### Results

#### Socio-demographic characteristics

Majority of the study participants 169 (60.6%) were males. The male to female ratio was 1.54:1. The age range was from 6 to 16 years with a mean age of 11 (SD ± 2.3) years. One-hundred sixty four (58.8%) study participants were in the age group of 11–15 years. Most of them 205 (73.3%) were urban resident and the family of 217 (77.8%) school children were literate (Table 1).

**Table 1:** Socio-demographic characteristics of primary school children at Haike primary school, Haike, North-East Ethiopia from April 2017 to May 2017 (n = 279)

Characteristics	Category	Number (%)
Age (years)	6–10	113 (40.5)
	11–15	164 (58.8)
	≥ 16	2 (0.7)
Sex	Male	169 (60.6)
	Female	110 (39.4)
Residence	Urban	205 (73.5)
	Rural	74 (26.5)
Family education	Literate	217 (77.8)
	Illiterate	62 (22.2)
Grade level	5–8 grade	122 (43.7)
	1–4 grade	157 (56.3)

Of the total examined primary school children, 52/279 (18.6%) and 85/279 (30.5%) were positive for intestinal parasite using direct wet mount and formol-ether concentration examination respectively (Additional file 2). The overall prevalence of single and double infections using formol-ether concentration technique was 82/279 (29.4%) and 3/279 (1.1%), respectively (Aditional file 3). The prevalence was higher in males 57 (33.7%) than females 28 (25.7%) (Table 2).

**Table 2:** Distribution of parasites among study participants at Haike primary school, Haike, North-East Ethiopia from April 2017 to May 2017

Type of parasites isolated	Wet mount			Formol-ether concentration technique		
	Total number (%)	Male (N = 169) N (%)	Female (N = 110) N (%)	Total number (%)	Male (N = 169) N (%)	Female (N = 110) N (%)
*S. mansoni*	44 (84.6)	30 (17.8)	14 (12.7)	65 (76.5)	48 (28.4)	17 (15.5)
*H. nana*	5 (9.6)	2 (1.2)	3 (2.7)	9 (10.6)	4 (2.4)	5 (4.5)
*E. vermicularis*	2 (3.9)	0 (0.0)	2 (1.8)	2 (2.4)	1 (0.6)	2 (1.8)
Hookworm	0 (0.0)	0 (0.0)	0 (0.0)	3 (3.5)	0 (0.0)	3 (2.7)
*Taenia* spps.	0 (0.0)	0 (0.0)	0 (0.0)	2 (2.4)	1 (0.6)	1 (0.9)
*E. histolytica*	1 (1.9)	1 (0.6)	0 (0.0)	1 (1.2)	0 (0.0)	1 (0.4)
*S. mansoni* and *H. nana*	0 (0.0)	0 (0.0)	0 (0.0)	1 (1.2)	1 (0.6)	0 (0.0)
*S. mansoni* and hookworm	0 (0.0)	0 (0.0)	0 (0.0)	1 (1.2)	0 (0.0)	1 (0.9)
*S. mansoni* and *E. histolytica*	0 (0.0	0 (0.0)	0 (0.0)	1 (1.2)	1 (0.6)	0 (0.0)
Total	52 (100.0)	33 (19.5)	19 (17.3)	85 (100.0)	56 (33.1)	29 (26.4)

*Schistosoma mansoni* was the dominant parasites by both direct wet mount and formol-ether concentration techniques with 44/52 (84.6%) and 65/85 (76.5%) respectively (Table 2).

In this study Chi square test showed a significant association between parasitic infection and age (p = 0.003). Furthermore, grade level had significant association with parasitic infection by direct wet mount examination but no by formol-ether concentration technique. Swimming and shoe wearing habit had significant association with parasitic infections (p > 0.05). There was also significant association between hand washing habits and parasitic infection when examined by wet mount (Additional file 4).

Binary logistic regression analysis showed that swimming habit had a strong association (p = 0.00) with S*. mansoni* and geo-helminthes infection (AOR = 6.61, 95% CI 3.31–13.12). Although there was no overall statistical significant association, family used lake as source of water showed statistically significant association (AOR = 5.435, 95% CI 2.97–12.32). Those who get water from river (AOR = 1.24, 95% CI 0.33–4.66) were also more likely to be infected with *S. mansoni* and geo-helminthes than those whose source is tap water (Table 3).

**Table 3:** The association of intestinal parasitic infection and pre-disposing risk factors at Haike primary school children, Haike, North-East Ethiopia from April 2017 to May 2017

Risk factors	Category	Intestinal parasitic infection		COR (95% CI)	p value	AOR (95% CI)	p value
		Positive [N (%)]	Negative [N (%)]				
Water container coverage	Always	83 (30.5)	189 (69.5)	1	0.91	1	
	No	2 (28.6)	5 (71.4)	1.10 (0.21–5.77)		2.11 (0.29–15.42)	0.50
Water source	Lake	2 (16.7)	10 (83.3)	2.16 (0.46–10.1)	0.33	5.35 (2.97–12.32)	0.03*
	River	5 (35.7)	9 (64.3)	0.78 (0.25–2.40)	0.66	1.24 (0.33–4.66)	0.79
	Spring	5 (45.5)	6 (54.5)	0.52 (0.15–1.75)	0.29	0.62 (0.14–2.75)	0.53
	Tape	73 (30.2)	169 (69.8)	1		1	
Hand washing habit	Always	65 (28.4)	164 (71.6)	1		1	
	Sometimes	13 (34.2)	25 (65.8)	0.76 (0.37–1.58)	0.46	1.01 (0.41–2.47)	0.98
	Never	7 (58.3)	5 (41.7)	0.28 (0.09–0.92)	0.04	0.41 (0.11–1.54)	0.19
Fishing	Yes	9 (40.9)	13 (59.1)	1.65 (0.68–4.02)	0.27	1.22 (0.45–3.32)	0.69
	No	76 (29.6)	181 (70.4)	1		1	
Swimming habit	Yes	59 (51.3)	56 (48.7)	5.59 (3. 21–9.75)	0.00	6.61 (3.31–13.12)	0.00*
	No	26 (15.9)	138 (84.1)	1		1	
Shoe wearing habit	Always	76 (29.6)	181 (70.4)	1		1	
	Sometimes	1 (12.50	7 (87.5)	2.94 (0.36–24.3)	0.32	0.69 (0.17–2.78)	0.60
	Never	8 (57.1)	6 (42.9)	0.32 (0.11–0.94)	0.04	1.77 (0.17–18.3)	0.63
Latrine presence	Yes	78 (29.8)	184 (70.2)	1	0.33	1	
	No	7 (41.2)	10 (58.8)	0.61 (0.22–1.65)		0.70 (0.20–2.40)	0.57
Latrine usage habit	Always	78 (29.9)	183 (70.1)	1		1	
	Sometimes	5 (35.7)	9 (64.3)	0.77 (0.25–2.36)	0.61	1.14 (0.10–11.94)	0.92
	Never	2 (50.0)	2 (50.0)	0.43 (0.06–3.08)	0.39	0.82 (0.06–11.54)	0.89

* Factors with statistically significance

### Discussion

*Schistosoma mansoni* and soil-transmitted helminthes infections are among the most prevalent public health problems in the world [1, 3]. The prevalence of *S. mansoni* and soil-transmitted helminthes was lower than reports from northwest and southwest, Ethiopia [1, 2, 12, 13]. This might be due to differences in environmental conditions such as temperature and humidity, socio-economic conditions and community awareness about parasitic diseases. Variations in study period, method of diagnosis and other factors can also be the reason. In the present study *S. mansoni* infection is the dominant parasite which was in agreement with reports from North-West and South-West Ethiopia [1, 12]. However, it was disagreed with a report from Chuahit [13]. In contrast to study in Zarima town, Ethiopia, in this study *H. nana* (3.2%) was the second most prevalent parasite [1].

Although the prevalence of *S. mansoni* was lower than other studies [1, 2], it was the most commonly isolated parasites in the study area. This might be due to the presence of lake and permanent streams near to the Haike primary school. School children may practice swimming, bathing, washing cloth and other activities. In the present study double infections were detected in three school children. In contrast to this finding the study conducted in Zarima town reported relatively high number of double and multiple infections in school children [1]. This might be due to the better hygienic practice of the school children and their family in our study area.

In current study the prevalence of *S. mansoni* and geo-helminthic infection had statistically significant association with the swimming habit and family source of water. This was in agreement with a study conducted in Congo [6]. *Schistosoma mansoni* and soil-transmitted helminthes infection were higher in males 57 (33.7%) than females 28 (25.7%). This finding might be due to the fact that males are usually engaged in swimming, fishing and other activities that might expose them for *S. mansoni* and other helminthic infections. However, this finding was not in agreement with a report from Congo which indicated similar prevalence in both gender [6].

In this study there was a statistically significant association between family used lake as source of water either for drinking or washing purpose and parasitic infection (AOR = 5.35, 95% CI 2.97–12.32). This might be due to the fact that lakes and streams are usually contaminated by human and animal excreta which result in parasite transmission when school children used the contaminated water for drinking and other purposes. In contrast to this finding, in the study conducted in Zarima town higher prevalence of parasitic infection were observed people who used protected spring as water source [1]. In addition, school children whose activities are fetching water and washing clothes in the lakes and river may have high vulnerability for parasitic infections.

### Conclusion and recommendation

*Schistosoma mansoni* and other helminthes are still important public health problems in the study area. *Schistosoma mansoni* infection was the pre-dominant parasitic infection in the school children. Health professionals especially health extension workers should teach school children, parents, school community about the risk of direct contact in Ketie river and about basic hygienic practices.

## Limitations

Due to lack of resources in this research Kato-katz technique was not performed which would help to estimate the intensity of *S. mansoni* and other helminthes infection. We recommend other researchers to investigate thoroughly using Kato-katz technique on the same study group as we found *S. mansoni* is the dominant helminthic parasite detected in the study area.

## Additional files

**Additional file 4.** Map of the study area ( Haike town).

**Additional file 1.** The prevalence of *S. mansoni* and other helminthes infection at Haike primary school, Haike, North-East Ethiopia from April 2017 to May 2017.

**Additional file 2.** Intestinal parasitic infection at different socio-demographic characteristics at Haike primary school children, Haike, North-East Ethiopia from April 2017 to May 2017.

**Additional file 3.** Intestinal parasitic infection at different associated risk factors at Haike primary school children, Haike, North-East Ethiopia from April 2017 to May 2017.

## Abbreviations

AOR: adjusted odds ratio
COR: crude odds ratio
g: gram
