# Supplementary material for: Schistosoma mansoni and other helminthes infections at Haike primary school children, North-East, Ethiopia: a cross-sectional study
Source: BMC Res Notes. 2017 Nov 21;10:609. doi: 10.1186/s13104-017-2942-9 (PMC5699180; doi:10.1186/s13104-017-2942-9)
Supplement: Supplementary file 2 — Additional file 1. The prevalence of S. mansoni and other helminthes infection at Haike primary school, Haike, North-East Ethiopia from April 2017 to May 2017. [file 13104_2017_2942_MOESM2_ESM.docx]

**The prevalence of *S. mansoni* and other helminthes infection at Haike primary school, Haike, North-East Ethiopia from April 2017- May 2017**

| Variables | Category | Number examined  (N (%)) | Diagnostic techniques | | | |
| --- | --- | --- | --- | --- | --- | --- |
|  |  |  | Wet mount | | Formol-ether concentration technique | |
|  |  |  | Positive  (N (%)) | Negative (N (%)) | Positive  (N (%)) | Negative  (N (%)) |
| Sex | Male | 169 (60.6) | 34 (20.1) | 135 (79.9) | 57 (33.7) | 112 (66.3) |
|  | Female | 110 (39.4) | 18 (16.4) | 92 (83.) | 28 (25.7) | 82 (74.5) |
| Age | 6-10 yrs | 113 (40.5) | 12 (10.6) | 101 (89.4) | 23 (20.4) | 90 (79.6) |
|  | 11-15yrs | 164 (58.8) | 40 (24.4) | 124 (75.6) | 62 (37.8) | 102 (62.2) |
|  | >15yrs | 2 (0.7%) | 0 (0.0) | 2 (100.0) | 0 (0.0) | 2 (1.0) |
| Residence | Urban | 205 (73.5) | 34 (16.6) | 171 (83.4) | 59 (28.8) | 146 (71.2) |
|  | Rural | 74 (26.5) | 18 (24.3) | 56 (75.5) | 26 (35.1) | 48 (64.9) |
| Family education | Literate | 217 (77.8) | 36 (16.6) | 181 (83.4) | 42 (26.8) | 115 (73.2) |
|  | Illiterate | 62 (22.2) | 16 (25.8) | 46 (74.2) | 43 (35.2) | 79 (64.8) |
| Grade level | 1-4 | 157 (56.3) | 22 (14.0) | 135 (86.0) | 60 (27.6) | 157 (72.4) |
|  | 5-8 | 122 (43.7) | 30 (24.6) | 62 (75.4) | 25 (40.3) | 37 (59.7) |
