# Supplementary material for: Schistosoma mansoni and other helminthes infections at Haike primary school children, North-East, Ethiopia: a cross-sectional study
Source: BMC Res Notes. 2017 Nov 21;10:609. doi: 10.1186/s13104-017-2942-9 (PMC5699180; doi:10.1186/s13104-017-2942-9)
Supplement: Supplementary file 3 — Additional file 2. Intestinal parasitic infection at different socio-demographic characteristics at Haike primary school children, Haike, North-East Ethiopia from April 2017 to May 2017. [file 13104_2017_2942_MOESM3_ESM.docx]

**Intestinal parasitic infection at different socio-demographic characteristics at Haike primary school children, Haike, North-East Ethiopia from April 2017 to May 2017**

| Variables | Category | Diagnostic techniques | | | | | |
| --- | --- | --- | --- | --- | --- | --- | --- |
|  |  | Wet mount | | P-value | Formol-ether concentration technique | | P-value |
|  |  | Positive  (N (%)) | Negative  (N (%)) |  | Positive  (N (%)) | Negative  (N (%)) |  |
| Sex | Male | 34 (20.1) | 135 (79.9) | 0.20 | 57 (33.7) | 112 (66.3) | 0.14 |
|  | Female | 18 (16.4) | 92 (83.) |  | 28 (25.7) | 82 (74.5) |  |
| Age | 6-10 yrs | 12 (10.6) | 101 (89.4) | 0.07 | 23 (20.4) | 90 (79.6) | 0.01**^*^** |
|  | 11-15yrs | 40 (24.4) | 124 (75.6) |  | 62 (37.8) | 102 (62.2) |  |
|  | >15yrs | 0 (0.0) | 2 (100.0) |  | 0 (0.0) | 2 (1.0) |  |
| Residence | Urban | 34 (16.6) | 171 (83.4) | 0.09 | 59 (28.8) | 146 (71.2) | 0.38 |
|  | Rural | 18 (24.3) | 56 (75.5) |  | 26 (35.1) | 48 (64.9) |  |
| Family education | Literate | 36 (16.6) | 181 (83.4) | 0.06 | 42 (26.8) | 115 (73.2) | 0.06 |
|  | Illiterate | 16 (25.8) | 46 (74.2) |  | 43 (35.2) | 79 (64.8) |  |
| Grade level | 1-4 grade | 22 (14.0) | 135 (86.0) | 0.01 | 60 (27.6) | 157 (72.4) | 0.15 |
|  | 5-8 grade | 30 (24.6) | 62 (75.4) |  | 25 (40.3) | 37 (59.7) |  |
