# Supplementary material for: Schistosoma mansoni and other helminthes infections at Haike primary school children, North-East, Ethiopia: a cross-sectional study
Source: BMC Res Notes. 2017 Nov 21;10:609. doi: 10.1186/s13104-017-2942-9 (PMC5699180; doi:10.1186/s13104-017-2942-9)
Supplement: Supplementary file 4 — Additional file 3. Intestinal parasitic infection at different associated risk factors at Haike primary school children, Haike, North-East Ethiopia from April 2017 to May 2017. [file 13104_2017_2942_MOESM4_ESM.docx]

**Intestinal parasitic infection at different associated risk factors at Haike primary school children, Haike, North-east Ethiopia from April 2017 to May 2017**

| Risk factors | Category | Diagnostic techniques | | | | | |
| --- | --- | --- | --- | --- | --- | --- | --- |
|  |  | Wet mount | | | Formol-ether concentration technique | | |
|  |  | Positive (N (%)) | Negative (N (%)) | P-value | Positive  (N (%)) | Negative (N (%)) | P-value |
| Water container coverage | Always | 51 (18.8) | 221 (81.2) | 1.00 | 83 (30.5) | 189 (69.5) | 1.00 |
|  | No | 1 I14.3) | 6 (85.7) |  | 2 (28.6) | 5 (71.4) |  |
| Water source | Lake | 2 (16.7) | 10 (83.3) | 0.08 | 2 (16.7) | 10 (83.3) | 0.51 |
|  | River | 1 (7.1) | 13 (92.9) |  | 5 (35.7) | 9 (64.3) |  |
|  | Spring | 5 (45.5) | 6 (54.5) |  | 5 (45.5) | 6 (54.5) |  |
|  | Tape | 44 (18.2) | 198 (81.8) |  | 73 (30.2) | 169 (69.8) |  |
| Hand washing habit | Always | 40 (17.5) | 189 (82.5) | 0.02^*^ | 65 (28.4) | 164 (71.6) | 0.08 |
|  | Sometimes | 6 (15.8) | 32 (84.2) |  | 13 (34.2) | 25 (65.8) |  |
|  | Never | 6 (50.0) | 6 (50.0) |  | 7 (58.3) | 5 (41.7) |  |
| Fishing | Yes | 7 (31.8) | 15 (68.2) | 0.15 | 9 (40.9) | 13 (59.1) | 0.33 |
|  | No | 45 (17.5) | 212 (82.5) |  | 76 (29.6) | 181 (70.4) |  |
| Swimming habit | Yes | 39 (33.9) | 76 (66.1) | 0.00**^*^** | 59 (51.3) | 56 (48.7) | 0.00**^*^** |
|  | No | 13 (7.9) | 151 (92.1) |  | 26 (15.9) | 138 (84.1) |  |
| Shoe wearing habit | Always | 46 (17.9) | 211 (82.1) | 0.26 | 76 (29.6) | 181 (70.4) | 0.04**^*^** |
|  | Never | 5 (35.70 | 9 (64.3) |  | 8 (57.1) | 6 (42.9) |  |
|  | Sometimes | 1 (12.5) | 7 (87.5) |  | 1 (12.50 | 7 (87.5) |  |
| Latrine presence | Yes | 47 (17.9) | 215 (82.1) | 0.33 | 78 (29.8) | 184 (70.2) | 0.41 |
|  | No | 5 (29.4) | 12 (70.1) |  | 7 (41.2) | 10 (58.8) |  |
| Latrine usage habit | Always | 46 (17.6) | 215 (82.4) | 0.21 | 78 (29.9) | 183 (70.1) | 0.68 |
|  | Sometimes | 5 (35.70 | 9 (64.3) |  | 5 (35.7) | 9 (64.3) |  |
|  | Never | 1 (25.0) | 3 (75.0) |  | 2 (50.0) | 2 (50.0) |  |
