# Supplementary material for: Schistosoma mansoni and other helminthes infections at Haike primary school children, North-East, Ethiopia: a cross-sectional study
Source: BMC Res Notes. 2017 Nov 21;10:609. doi: 10.1186/s13104-017-2942-9 (PMC5699180; doi:10.1186/s13104-017-2942-9)
Supplement: Supplementary file 4 [file 13104_2017_2942_MOESM1_ESM.docx]

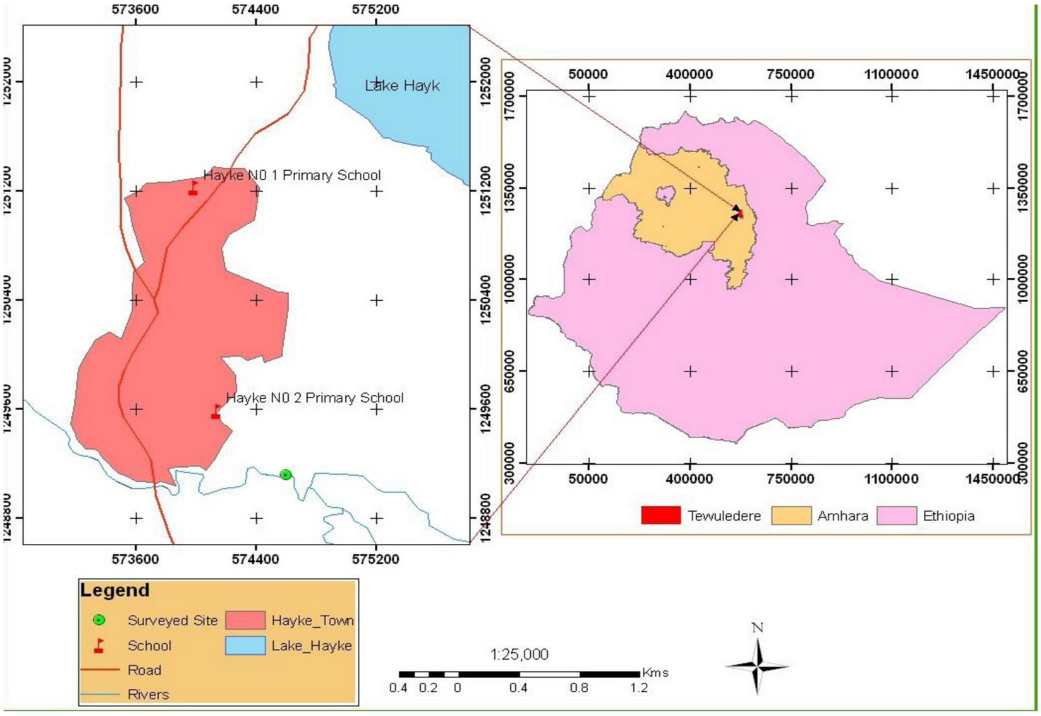

573600
574400
575200
1252000
1251200
1250400
1249600
1248800
Lake Hayk
Hayke N0 1 Primary School
Hayke N0 2 Primary School
50000
400000
750000
1100000
1450000
1700000
1350000
1000000
650000
300000
Tewuledere
Amhara
Ethiopia
Legend
Surveyed Site
School
Road
Rivers
Hayke_Town
Lake_Hayke
1:25,000
0.4 0.2 0 0.4 0.8 1.2
Kms
N
